# The effects of exercise on pain, fatigue, insomnia, and health perceptions in patients with operable advanced stage rectal cancer prior to surgery: a pilot trial

**DOI:** 10.1186/s12885-017-3130-y

**Published:** 2017-02-23

**Authors:** Jennifer Brunet, Shaunna Burke, Michael P.W. Grocott, Malcolm A. West, Sandy Jack

**Affiliations:** 1 Faculty of Health Sciences, School of Human Kinetics, University of Ottawa, 125 University Private, Montpetit Hall Room 339, Ottawa, ON K1N 6N5 Canada; 2 Institut de Recherche de l’Hôpital Montfort (IRHM), Hôpital Montfort, Ottawa, ON Canada; 3 Cancer Therapeutic Program, Ottawa Hospital Research Institute (OHRI), Ottawa, ON Canada; 4 Centre for Sport and Exercise Sciences, School of Biomedical Sciences, University of Leeds, Leeds, UK; 5 Integrative Physiology and Critical Illness Group, Clinical and Experimental Sciences, Faculty of Medicine, University of Southampton, Southampton, UK; 6 Academic Unit of Cancer Sciences, Faculty of Medicine, University of Southampton, Southampton, UK; 7 Critical Care Research Area, Southampton NIHR Respiratory Biomedical Research Unit, Southampton, UK; 8 Anaesthesia and Critical Care Research Unit, University Hospital Southampton NHS Foundation Trust, Southampton, UK

**Keywords:** Rectal cancer, Advanced stage, Exercise, Experimental study design, Patient-reported outcomes, Quality of life

## Abstract

**Background:**

Promoting quality of life (QoL) is a key priority in cancer care. We investigated the hypothesis that, in comparison to usual care, exercise post-neoadjuvant chemoradiation therapy/prior to surgical resection will reduce pain, fatigue, and insomnia, and will improve physical and mental health perceptions in patients with locally advanced stage rectal cancer.

**Methods:**

In this non-randomized controlled pilot trial, patients in the supervised exercise group (EG; *M*_age_ = 64 years; 64% male) and in the control group (CG; *M*_age_ = 72 years; 69% male) completed the European Organization for Research and Treatment of Cancer core Quality of Life questionnaire and the RAND 36-Item Health Survey three times: pre-neoadjuvant chemoradiation therapy (Time 1; *n*_EC_ = 24; *n*_CG_ = 11), post-neoadjuvant chemoradiation therapy/pre-exercise intervention (Time 2; *n*_EC_ = 23; *n*_CG_ = 10), and post-exercise intervention (Time 3; *n*_EC_ = 22; *n*_CG_ = 10). The 6-week exercise intervention was delivered in hospital and comprised of interval aerobic training. Patients trained in pairs three times per week for 30 to 40 min. Data were analyzed by Mann–Whitney tests and by Wilcoxon matched-pairs signed-rank tests.

**Results:**

No significant between-group differences in changes were found for any of the outcomes. In both groups, fatigue levels decreased and physical health perceptions increased from pre- to post-exercise intervention. Pain levels also decreased from pre- to post-exercise intervention, albeit not significantly.

**Conclusions:**

The findings from this study can be used to guide a more definitive trial as they provide preliminary evidence regarding the potential effects of pre-operative exercise on self-reported pain, fatigue, insomnia, and health perceptions in patients with locally advanced rectal cancer. Trial registration: This study has been registered with clinicaltrials.gov (NCT01325909; March 29, 2011).

## Background

Approximately 813,613 men and 663,689 women were diagnosed with rectal cancer worldwide in 2012 [1]. Of these, 50–65% were diagnosed with locally advanced rectal cancer. Treatment for locally advanced rectal cancer often involves neoadjuvant chemoradiation therapy followed by surgical resection with the aim of improving resectability and disease control [2]. Although these standard treatments can prolong survival, they can result in adverse physical side effects, including pain, fatigue, constipation or diarrhea, upset stomach, nausea, sexual problems, infertility, acute toxicity, and decreased physical fitness [3, 4]. They can also result in adverse psychological side effects, including anxiety and distress [5]. As a result of these treatment-related side effects, patients’ quality of life (QoL) is often impaired [6]. Considering that QoL is a significant prognostic factor for cancer recurrence and all-cause mortality in patients with advanced colorectal cancer [7], identifying therapies to reduce treatment-related side effects and enhance QoL is a priority in the care of patients with advanced rectal cancer.

Exercise is one type of therapy that may improve outcomes for patients with advanced cancer at different stages of the disease trajectory. For example, researchers have reported that *post-operative* exercise can prolong survival after cancer diagnosis [8, 9], as well as enhance QoL by helping patients with advanced stage cancer manage physical and psychological side effects [10]. In addition, researchers have reported that *pre-operative* exercise is beneficial for patients with colorectal [11], colon [12, 13], and rectal cancer [14]. Specifically, they have shown that it can improve cardiorespiratory fitness [14], muscle strength [14], peak power output [13], heart rate [13], oxygen uptake [13], and respiratory muscle endurance [12]. This provides evidence that pre-operative exercise can elicit favourable changes in physiological outcomes in patients with advanced stage cancer [15, 16]. However, limited data are currently available to determine the effects of exercise post-neoadjuvant chemoradiation therapy and prior to surgical resection on key patient-reported outcomes (e.g., pain, fatigue, insomnia, health perceptions) in patients with advanced rectal cancer. Considering that advanced rectal cancer and neoadjuvant chemoradiation therapy can adversely affect patients’ general physical and mental health perceptions and increase fatigue, pain, and insomnia [17, 18], which can negatively affect recovery [5], it is important to examine whether participating in pre-operative exercise can help prevent or reduce these adverse consequences reported by patients.

### The present study

We delivered a 6-week exercise intervention to patients diagnosed with locally advanced rectal cancer immediately post-neoadjuvant chemoradiation therapy and prior to surgical resection in order to examine the benefits of exercise at this particular stage of the disease trajectory. We examined changes in various patient-reported outcomes resulting from the exercise intervention using quantitative and qualitative methods. The aim of our qualitative inquiry was to capture in-depth accounts of changes in QoL associated with the exercise intervention from patients’ perspectives [19]. We had several aims in mind for our quantitative inquiry. Herein, we focus on the two aims related to changes in QoL. The first aim was to assess the effects of the exercise intervention on indicators of QoL in comparison to usual care (i.e., assess differences in changes between groups). The second aim was to quantify the extent to which the exercise intervention had a positive effect on indicators of QoL (i.e., assess within-group changes). We focused on pain, fatigue, insomnia, and physical and mental health perceptions as indicators of QoL because (i) patients with rectal cancer report these as main concerns [17], (ii) these symptoms appear in the National Institute of Health call for more efforts toward symptom management in cancer [20], and (iii) they represent different dimensions of health relevant to patients with cancer [21].

## Methods

Data analyzed for this study were collected as part of a single-site, non-randomized controlled pilot trial. We have published analyses using this sample elsewhere [22, 23]. Additional details of the methods that are not relevant to this study can be found in those publications. The protocol was approved by the North West – Liverpool East Committee for Research Ethics (11/H1002/12) and it was registered with clinicaltrials.gov (NCT01325909; March 29, 2011). Patients provided informed consent to participate in this study prior to us conducting any study-related procedures.

### Participants and procedures

From March 2011 to February 2013, patients referred to the colorectal multidisciplinary team were recruited for this study. Inclusion criteria were: (i) ≥ 18 years of age, (ii) confirmed diagnosis of magnetic resonance imaging defined locally advanced circumferential margin threatened resectable rectal cancer (i.e., ≥ stage T2/N+ with no distant metastasis), (iii) scheduled for standardized neoadjuvant chemoradiation therapy, and (iv) performance status score of ≤ 2 on the Eastern Co-operative Oncology Group (ECOG)/World Health Organization (WHO) system [24]. Patients were not eligible if they: (i) were unable to give informed consent, (ii) had been diagnosed with non-resectable cancer, (iii) were unable to perform a cardiopulmonary exercise test (CPET) or exercise, (iv) had declined surgery or neoadjuvant chemoradiation therapy, and/or (v) had received non-standard neoadjuvant chemoradiation therapy.

All patients in this study underwent 5 weeks of standardized neoadjuvant chemoradiation therapy. Standardized radiotherapy consisted of 45 Gray (Gy) in 25 fractions on weekdays using a three-dimensional conformal technique with computerized tomography guidance. A booster dose was given (5.4 Gy in 3 fractions) to the primary tumour only. Oral capecitabine at a dose of 825 mg.m^−2^ was given twice daily on radiotherapy days. No patient received brachytherapy.

**Fig. 1:**
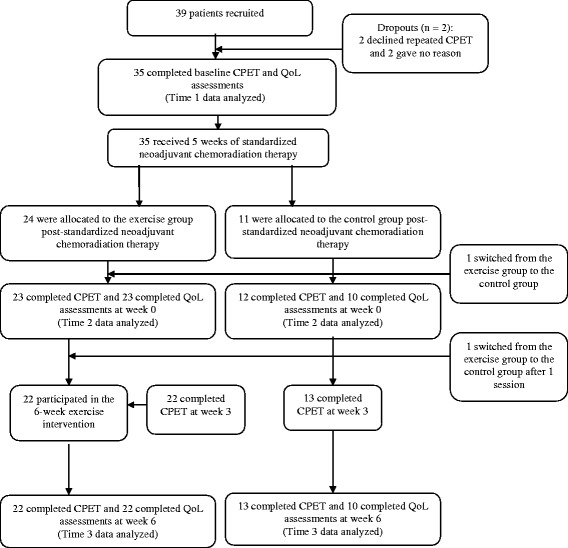
Flow chart of recruitment and participation in this study

After completing neoadjuvant chemoradiation therapy, all patients were assigned to the exercise group by default (i.e., there was no allocation concealment) by the colorectal multidisciplinary team unless they were unable to commit to the exercise schedule or lived > 15 miles from the hospital. These latter patients were asked to act as contemporaneously recruited controls. A total of 39 patients were recruited into the study, though four dropped out immediately. Thirty-five patients completed QoL assessments prior to receiving neoadjuvant chemoradiation therapy (Time 1 data analyzed) and went on to receive neoadjuvant chemoradiation therapy. Thereafter, 24 were allocated to the exercise group and 11 to the control group, though 1 patient switched immediately to the control group. At this time, 23 patients in the exercise group and 10 patients in the control group completed QoL assessments prior to the 6-week exercise intervention (Time 2 data analyzed). After the exercise intervention, 22 patients remained in the exercise group and completed QoL assessments along with 10 patients in the control group (Time 3 data analyzed). Figure 1 displays the flow of patients through each stage of this study from enrolment to analysis. We note that the sample size for analysis herein is slightly different from previous publications [22, 23] due to the completeness of relevant data (i.e., the previous publications used CPET data and the current study used QoL data).

### Study procedures

#### Assessments

Patients completed questionnaires prior to neoadjuvant chemoradiation therapy (Time 1), before starting the exercise intervention (i.e., immediately post-neoadjuvant chemoradiation therapy; Time 2), and immediately post-exercise intervention (Time 3). They also underwent a standardized CPET to assess their cardiovascular, respiratory, and skeletal muscle systems (see [25] for protocol details) at these three time points^1^; however, an additional CPET was performed mid-way through the exercise intervention so as to modify the exercise prescription according to patients’ changing fitness levels. Prior to receiving the exercise intervention, patients received usual care from their oncology care team.

#### Exercise intervention

The exercise protocol was progressive and lasted 6 weeks. Patients exercised in pairs three times per week under the supervision of a trained exercise specialist in a hospital. Initially, exercise intensities were tailored for each patient based on his/her standardized CPET results post-chemoradiation therapy and modified thereafter according to his/her results mid-way through the exercise intervention. Each patient was instructed to engage in interval training on an electromagnetically braked cycle ergometer (Optibike Ergoline GmbH, Germany). A chip-and-pin card with patients’ pre-loaded target interval intensities was used to ensure they engaged in 3 min of moderate-intensity intervals (i.e., work rate of 80% of oxygen uptake at lactate threshold) interspersed with 2 min of vigorous-intensity intervals (i.e., work rate of 50% of the difference in work rates between peak oxygen uptake and oxygen uptake at lactate threshold). For the first three sessions, training consisted of a total time of 30 min, which was then increased to 40 min for the rest of the training sessions. All sessions included 5 min of warm-up and 5 min of cool-down.

### Outcome measures

At each of the three time points, we used the European Organization for Research and Treatment of Cancer 30-item core Quality of Life questionnaire (EORTC QOL-C30) version 3 [26] to assess patients’ levels of pain, fatigue, and insomnia, and used the RAND 36-Item Health Survey [27] to assess their general physical and mental health perceptions.

The EORTC QLQ-C30 is a self-report questionnaire developed to assess cancer patients’ QoL. It comprises five multi-item functional subscales (i.e., role, physical, cognitive, emotional, and social functioning), three multi-item symptom scales (i.e., fatigue, pain, and nausea), five single items assessing common symptoms experienced (i.e., dyspnea, insomnia, appetite loss, constipation, and diarrhea), and two questions assessing global health status/QoL. Each item has four response options: (1) *not at all*, (2) *a little*, (3) *quite a bit*, and (4) *very much*, except for the two questions assessing global health status/QoL [response options range from (1) *very poor* to (7) *excellent*]. Higher scores on the functional subscales and global health status/QoL scale represent a better level of functioning and global health status/QoL, whereas higher scores on symptom subscales represent higher levels of symptomatology. Given that cancer and neoadjuvant chemoradiation therapy can increase fatigue, pain, and insomnia [17, 18], which can negatively affect recovery [5], these scales were the focus of the current analyses.

The RAND 36-Item Health Survey is a self-report questionnaire that consists of eight subscales assessing the health domains of physical functioning, social functioning, role limitations due to physical health problems, role limitations due to emotional health problems, vitality/energy, bodily pain, general health perceptions, and mental health perceptions. It includes the same items as those in the 36-item Short-Form (SF-36) Health Survey [28]; however, each item is scored on a scale ranging from 0 to 100. Scores on the physical functioning, role limitations due to physical health problems, bodily pain, and general health perceptions subscales were averaged into a physical component summary score. Scores on the social functioning, role limitations due to emotional health problems, vitality/energy, and mental health subscales were averaged into a mental component summary score. Higher scores represent better physical and mental health perceptions.

### Statistical analysis

All statistical analyses were performed using SPSS version 23 and included all data available at any given point. Missing values were not imputed for analysis. Descriptive data were used to describe differences on the QoL measures across time points, and are expressed as medians and inter-quartile ranges at each time point. As the distribution of the variables was significantly different from normal based on Kolmogorov-Smirnov tests for three variables (i.e., pain, insomnia, and mental health perceptions), non-parametric tests were used. Specifically, Mann–Whitney tests were used to assess whether changes in fatigue, pain, insomnia, and health perceptions across time points differed between the exercise group and the control group (i.e., Aim 1; assess differences in changes between the two groups). Wilcoxon matched-pairs signed-rank tests were used to identify any changes in fatigue, pain, insomnia, and health perceptions across time points within-groups (i.e., Aim 2; assess within-group changes). Of note, testing using parametric tests (i.e., *t*-tests) for variables with normal distributions yielded results similar to those obtained with the non-parametric tests (data not shown). To correct for multiple comparisons, we used the Simes procedure [29] – a modification of the Bonferroni correction method. Accordingly, level of statistical significance was set to *p* < .017.

## Results

Patients in the exercise group had a mean age of 64 years (range = 45 – 82), 64% were male, and they had a mean body mass index of 27.4 kg/m^2^ (*SD* = 5.1). Forty-five percent were currently smoking, and 46% had a past medical history of diabetes, health failure, or ischemic heart disease. Most (82%) scored ‘0’ on the ECOG/WHO system meaning that they were asymptomatic (i.e., fully active and able to carry on all pre-disease activities without restriction). The rest (18%) scored ‘1’ meaning they were symptomatic but completely ambulatory (i.e., restricted in physically strenuous activity but ambulatory and able to carry out work of a light or sedentary nature). No patient scored ‘2’ meaning none were symptomatic (i.e., <50% in bed during the day, ambulatory and capable of all self care but unable to carry out any work activities, and up and about  >  50% of waking hours). Overall, patients adhered well to the exercise protocol, as the mean (*SD*) attendance for the patients who took part in the exercise intervention was 96% (5.0). There were no adverse events reported.

Patients in the control group had a mean age of 72 years (range = 62 – 84), 69% were male, and they had a mean body mass index of 24.9 kg/m^2^ (*SD* = 3.9). Thirty-one percent were currently smoking, and 54% had a past medical history of diabetes, health failure, or ischemic heart disease. Most (62%) scored ‘0’ on the ECOG/WHO, 23% scored ‘1’, and 15% scored ‘2’.

### Aim 1: Examining differences in changes between groups

There was no evidence that changes in pain (*p* = .67), fatigue (*p* = .10), insomnia (*p* = .89), physical health perceptions (*p* = .34), and mental health perceptions (*p* = .90) observed from pre- to post-exercise intervention differed significantly between the exercise group and the control group.

### Aim 2: Examining within-group changes

Prior to neoadjuvant chemoradiation therapy, median scores of pain, fatigue, and insomnia were 17.0, 22.0, and 33.0 for the total sample, respectively, which are comparable to published norms [30]. Median scores were 52.8 and 56.8 for physical and mental health perceptions, respectively, which also fall close to normative values [31]. Descriptive statistics for all outcomes for the exercise group and the control group by time point are presented in Table 1.

**Table 1:** Summary of scores for each group by time point expressed as medians and inter-quartile ranges

	Pain	Fatigue	Insomnia	Physical health	Mental health
Baseline					
Control (*n =* 11)	0 (0,33.0)	11.0 (11.1,44.0)	0 (0,33.0)	52.8 (32.8,64.4)	59.0 (53.3,63.4)
Intervention (*n =* 24)	16.7 (0,33.3)	27.5 (11.0,50.3)	33.3 (0,67.0)	53.1 (33.5,63.1)	56.0 (51.3,63.4)
Total *(n =* 35)	17.0 (0,33.0)	22.0 (11.0,44.0)	33.0 (0,67.0)	52.8 (33.0,63.4)	56.8 (51.8,63.4)
Pre-exercise intervention					
Control (*n =* 10)	33.0 (29.0,62.5)	33.0 (19.3,49.8)	33.0 (0,42.5)	29.6 (24.4,34.7)	52.0 (43.3,61.3)
Intervention (*n =* 23)	33.0 (17.0,50.0)	33.0 (22.0,67.0)	33.0 (33.0,67.0)	39.2 (26.6,55.2)	57.0 (51.3,62.4)
Total *(n =* 33)	33.0 (17.0,50.0)	33.0 (22.0,67.0)	33.0 (16.5,67.0)	36.4 (26.4,53.3)	55.0 (50.3,61.7)
Post-exercise intervention					
Control (*n =* 10)	8.5 (0,41.5)	22.0 (0,35.8)	0 (0,49.8)	56.8 (30.7,64.1)	55.1 (51.2,58.6)
Intervention (*n =* 22)	8.5 (0,37.3)	22.0 (11.0,33.0)	33.0 (0,67.0)	57.3 (37.3,63.1)	56.1 (53.5,60.7)
Total *(n =* 32)	8.5 (0,33.0)	22.0 (2.3,33.0)	16.5 (0,67.0)	57.3 (37.1,63.3)	55.5 (53.0,59.5)

#### Pain

There were changes in levels of pain from pre- to post-neoadjuvant chemoradiation therapy (*p*s < .03), wherein patients in both groups reported more pain immediately post-neoadjuvant chemoradiation therapy compared to pre-neoadjuvant chemoradiation therapy. Whereas patients in both groups reported less pain post-exercise intervention, these were not statistically different from those pre-exercise intervention (*p*s > .14).

#### Fatigue

There were changes in levels of fatigue from pre- to post-neoadjuvant chemoradiation therapy (*p*s *<* .001) and from pre- to post-exercise intervention (*p*s < .01). Specifically, patients in both groups reported more fatigue immediately post-neoadjuvant chemoradiation therapy compared to pre-neoadjuvant chemoradiation therapy, and reported less fatigue post-exercise intervention compared to pre-exercise intervention.

#### Insomnia

There were changes in levels of insomnia for patients in the control group from pre- to post-neoadjuvant chemoradiation therapy (*p* = .05) and from pre- to post-exercise intervention (*p* = .04), albeit not significantly based on the corrected critical *p*-value. These patients reported more insomnia immediately post-neoadjuvant chemoradiation therapy compared to pre-neoadjuvant chemoradiation therapy, and reported less insomnia post-exercise intervention compared to pre-exercise intervention. There were no significant differences in levels of insomnia across time points (*p*s ≥ .26) for patients in the exercise group.

#### Physical health

There were changes in physical health perceptions from pre- to post-neoadjuvant chemoradiation therapy (*p*s < .007) and from pre- to post-exercise intervention (*p*s < .004). Patients in both groups reported poorer physical health perceptions immediately post-neoadjuvant chemoradiation therapy compared to pre-neoadjuvant chemoradiation therapy, and better physical health perceptions post-exercise intervention compared to pre-exercise intervention.

#### Mental health

There were no changes in mental health perceptions across time points for either of the groups (*p*s ≥ .43).

## Discussion

The wait period between the completion of neoadjuvant chemoradiation therapy and prior to surgery can be challenging for patients with advanced rectal cancer. Debilitating side effects can impair recovery and reduce QoL in this population [5]. Yet, relatively few studies have been conducted to examine whether pre-operative exercise is an effective approach to help patients manage treatment-related side effects and promote QoL during this time. In this study, we explored the effects of a 6-week exercise intervention on pain, fatigue, insomnia, and health perceptions in patients with locally advanced cancer who had recently completed neoadjuvant chemoradiation therapy.

We found no evidence that an exercise intervention delivered in hospital and that comprised of interval aerobic training resulted in greater effects for any of the outcomes in comparison to usual care, and thus failed to support the notion that this type of exercise intervention is more effective than usual care for reducing treatment-related side effects and improving QoL. However, it is important to note that our study procedures may explain these findings. In the current study, all patients were assigned to the exercise group by default, unless they were unable to commit to the exercise schedule or lived > 15 miles from the hospital. In retrospect, presenting patients in the control group with the exercise intervention could have prompted them to reflect on their current behaviour, made them recognize that there is a need to change their behaviour, and in some cases, led them to make changes to it. Indeed, patients in both groups increased their average number of steps from pre- to post-exercise intervention (see [22], Figure 4). Thus, this may have led to an under-estimation of the effects of the exercise intervention in comparison to usual care. With this in mind, we believe that there are potentially some patients that may not need this type of pre-operative intervention to manage their treatment-related side effects and improve their QoL as they may be active on their own. Observed improvements for the control group may also be explained by other factors. For example, those in the control group may have sought other types of treatments (e.g., pharmaceuticals, psychological therapy, group therapy), which could have had positive effects on the outcomes we assessed. To control for this, we recommend conducting a randomized controlled trial in which participation in various therapies and exercise is measured and controlled for. We are currently conducting a randomized controlled trial (NCT01914068) in order to mitigate these study design limitations.

Whilst our findings do not support the notion that this type of exercise intervention is more effective than usual care in reducing treatment-related side effects and improving QoL, they demonstrate the likely value of exercise post-neoadjuvant chemoradiation therapy/prior to surgery for patients with advanced rectal cancer. This is because we observed a significant improvement in physical health perceptions and a decrease in levels of fatigue post-exercise intervention for patients in the exercise group. Moreover, we noted decreases in levels of pain post-exercise intervention for these patients, though these did not reach statistical significance.

Previous observational and experimental studies have demonstrated that post-operative exercise reduces fatigue in adults with cancer [10, 32]. Our findings extend these observations, demonstrating that a pre-operative exercise intervention can decrease fatigue – which happens to be the most frequent symptom cited [17] – in a group of patients who had completed neoadjuvant chemoradiation therapy for advanced stage rectal cancer. This finding is important when considering that patients’ levels of fatigue significantly increased after neoadjuvant chemoradiation therapy, and that fatigue can negatively affect QoL more than any other symptom such as vomiting, nausea, pain, and depression [33, 34]. While the exact process through which exercise reduced patients’ levels of fatigue remains to be determined, it could be that it helped to restore their physical capacity and fitness [35]. Indeed, for patients in the exercise group, their oxygen uptake at lactate threshold significantly improved post-exercise intervention (data reported elsewhere; [22]). Thus, future research attempting to determine which aspects of pre-operative exercise helps to reduce fatigue would be beneficial to optimize pre-operative exercise interventions aimed at reducing fatigue in this population.

Although we did not observe a statistically significant difference in change between groups, we observed that exercise post-neoadjuvant chemoradiation therapy significantly improved patients’ physical health perceptions. This finding is consistent with previous studies in which patients receiving treatment for either a primary, recurrent incurable cancer or advanced cancer showed improvements in health perceptions post-exercise [36–38]. These findings are significant because decreases in physical health are common during the post-neoadjuvant chemoradiation therapy period [3, 33, 34] and lead to more adverse surgical outcomes (e.g., prolonged hospital stay; [39]). Moreover, this may have clinical significance because self-rated health is a significant predictor of survival in adults with advanced cancer [40].

Though our results suggest that our exercise intervention did not have a statistically significant effect on pain, these should be interpreted cautiously. The non-significant trend for patients to report less pain post-exercise intervention as compared to pre-exercise intervention may have been the result of insufficient power. Hence, it is necessary to keep in mind that patients’ levels of pain decreased post-exercise intervention, and that they were lower than their pre-neoadjuvant chemoradiation therapy levels. Further, compared to reference data published for patients with rectal cancer [30], patients in this study reported lower levels of pain post-exercise intervention. Thus, it is recommended that studies with larger samples sizes be conducted to assess the extent to which exercise may have an impact on pain during this time in this population.

In contrast to previous research that suggests exercise can reduce anxiety, depression, and sleep disturbances during and post-treatment in adults with cancer [38], we did not find statistically significant improvements in insomnia or mental health perceptions. Neither insomnia nor mental health perceptions worsened during neoadjuvant chemoradiation therapy, and levels were comparable to normative levels [30]. This may have left less room for improvement than if patients had high levels of insomnia and poor mental health perceptions after undergoing neoadjuvant chemoradiation therapy. Alternatively, the non-significant effects of exercise on these outcomes might be due to the short duration of our intervention (i.e., 6 weeks). Based on previous reports [41], longer interventions might be necessary to change mental health perceptions and insomnia. Patients could have also been taking pharmaceuticals or have received psychological therapy (data not collected) to manage their insomnia and/or mental health issues [42], which may have confounded the effects of exercise on these outcomes. Last, the measures used, though valid and reliable, might not have been sensitive enough to capture changes in these two patient-reported outcomes. For instance, insomnia was only measured using one item, which may fail to capture insomnia symptoms along several dimensions (i.e., severity, duration, and impact). Assessing insomnia using questionnaires that capture the nature, severity, and impact of insomnia may be more effective for determining if exercise has an impact on insomnia. As well, previous studies have shown that adults with cancer are likely to experience unanticipated fear, anxiety, and psychological stress about major surgery [5]. The mental health summary score derived from the RAND 36-Item Health Survey might not be sensitive to measuring these specific cancer-related mental health issues (e.g., pre-operative anxiety) that might have been affected by exercise. These possible explanations should be investigated in future research.

### Limitations

Perhaps the most significant limitation of this study is the small sample size of the control group that could have introduced Type II error when testing for differences between the exercise group and the control group. Indeed, power calculations were only made to determine the sample size required to detect a minimum difference in oxygen uptake at lactate threshold of 1.5 ml kg^−1^ min^−1^ and a *SD* of 1.1 ml kg^−1^ min^−1^ [22], not QoL. Relatedly, because the sample size was small and the data were not normally distributed for three variables, non-parametric statistical tests that do not require the assumptions of normality be met were used. However, it should be noted that non-parametric tests are more conservative and are appropriate for hypothesis testing when the sample size is small. Other limitations include the reliance on a convenience sample, our inability to report the rate of recruitment because the number of patients eligible was not recorded, and the non-randomization. The latter increases the likelihood of there being differences between the exercise group and the control group in factors (known and unknown) that could affect the outcomes we assessed. Also, this study has the potential for ascertainment bias due to the fact that patients were given a choice to participate in the exercise intervention. Consequently, the effects observed may be biased upwards. A final limitation is the lack of follow-up data to determine if the observed improvements were maintained over time and whether pre-operative exercise reduced the incidence of post-operative complications. Thus, a larger, adequately powered randomized controlled trial with long-term follow-ups is needed to compare the effects of exercise post-neoadjuvant chemoradiation therapy/prior to surgical resection on pain, fatigue, insomnia, and physical and mental health perceptions, in comparison to usual care, in patients with locally advanced stage rectal cancer.

## Conclusions

Pain, fatigue, and insomnia are prevalent and disturbing side effects of treatment for advanced rectal cancer. Furthermore, treatment for advanced rectal can result in diminished health perceptions and QoL. The notion that exercise has a greater effect on self-reported pain, fatigue, insomnia, and health perceptions than usual care was not confirmed in this study. Nevertheless, we did observe an increase in physical health perceptions and a decrease in levels of fatigue post-exercise intervention for patients in the exercise group. We also found small, but not statistically significant, decreases in levels of pain post-exercise intervention for these patients. In light of the limitations associated with this study, it is important that a larger randomized controlled trial be conducted to assess the effectiveness of exercise in comparison to usual care, and to provide precise estimates of the effects of exercise on key patient-reported outcomes. Such a study would provide valuable insight into the extent to which pre-operative exercise is effective in treating patients’ side effects and promoting improvements in the quality of their lives above and beyond usual care.

## Abbreviations

CG: Control group
CPET: Cardiopulmonary exercise test
ECOG: Eastern Co-operative Oncology Group
EG: Exercise group
EORTC QOL-C30: European Organization for Research and Treatment of Cancer 30-item core Quality of Life questionnaire
Gy: Gray
QoL: Quality of life
SF-36: 36-item Short-Form Health Survey
WHO: World Health Organization

## Footnotes

1 Changes in objectively-measured physical fitness are reported elsewhere [22, 23].
